# Phosphate Concentration and Arbuscular Mycorrhizal Colonisation Influence the Growth, Yield and Expression of Twelve *PHT1* Family Phosphate Transporters in Foxtail Millet (*Setaria italica*)

**DOI:** 10.1371/journal.pone.0108459

**Published:** 2014-09-24

**Authors:** S. Antony Ceasar, Angela Hodge, Alison Baker, Stephen A. Baldwin

**Affiliations:** 1 Centre for Plant Sciences and School of Molecular and Cellular Biology, Faculty of Biological Sciences, University of Leeds, Leeds, United Kingdom; 2 Astbury Centre for Structural Molecular Biology and School of Biomedical Sciences, Faculty of Biological Sciences, University of Leeds, Leeds, United Kingdom; 3 Department of Biology, University of York, Wentworth Way, York, United Kingdom; University of Tartu, Estonia

## Abstract

Phosphorus (P) is an essential element which plays several key roles in all living organisms. *Setaria italica* (foxtail millet) is a model species for panacoid grasses including several millet species widely grown in arid regions of Asia and Africa, and for the bioenergy crop switchgrass. The growth responses of *S. italica* to different levels of inorganic phosphate (Pi) and to colonisation with the arbuscular mycorrhizal fungus *Funneliformis mosseae* (syn. *Glomus mosseae*) were studied. Phosphate is taken up from the environment by the PHT1 family of plant phosphate transporters, which have been well characterized in several plant species. Bioinformatic analysis identified 12 members of the *PHT1* gene family (*SiPHT1;1-1;12*) in *S. italica*, and RT and qPCR analysis showed that most of these transporters displayed specific expression patterns with respect to tissue, phosphate status and arbuscular mycorrhizal colonisation. *SiPHT1;2* was found to be expressed in all tissues and in all growth conditions tested. In contrast, expression of *SiPHT1;4* was induced in roots after 15 days growth in hydroponic medium of low Pi concentration. Expression of *SiPHT1;8* and *SiPHT1;9* in roots was selectively induced by colonisation with *F. mosseae*. *SiPHT1;3* and *SiPHT1;4* were found to be predominantly expressed in leaf and root tissues respectively. Several other transporters were expressed in shoots and leaves during growth in low Pi concentrations. This study will form the basis for the further characterization of these transporters, with the long term goal of improving the phosphate use efficiency of foxtail millet.

## Introduction

Phosphorus (P) is an essential, non-substitutable element for plant growth. It is a component of cell membranes as phospholipids, and is involved in a multitude of functions including energy transfer, photosynthesis, many aspects of metabolism, intracellular signalling and gene replication and expression. Low availability of inorganic P (Pi) is a major constraint for crop production in many low-input agricultural systems worldwide [1]. The importance of phosphate for plant growth and yield has been reported in several crop species including barley [2], [3], maize [4], [5], sugar beet [6], common bean [7] and wheat [8]. Hence the acquisition and utilization of P by plants is a significant factor in the determination of final crop yield [9] and consequently P deficiency limits plant growth and crop productivity in many soils [10].

In traditional agricultural systems, farmers depend on the inherent fertility of the soil or the addition of manures to supply P for the crop. However, agriculture intensification has resulted in dependency on the application of phosphate fertilizers to increase crop yields [11]. Phosphate fertilizer is largely derived from rock phosphate, which is also the only significant global reserve of Pi and a non-renewable resource which, according to some estimates, maybe exhausted in only 50 to 100 years [12]. Clearly it would be advantageous to reduce the dependency of crops on external fertilizer addition without overly compromising yields. As membrane transporters are the means by which nutrients enter and are transported between cells, a fuller understanding of their roles and functions will be important for developing plants with improved phosphate acquisition and use efficiency [13].

Millets are an important group of plants predominantly cultivated and consumed by people in Asia and Africa. The seeds of millets are rich in essential nutrients including calcium, magnesium and iron, and are used as a major source of food for millions of people. The improvement of millets using biotechnological tools is crucial for strengthening the food security of poor people living in less developed nations [14]. Foxtail millet (*Setaria italica*), probably first cultivated some 8,000 years ago in China, is widely grown as a grain crop not only in the semi-arid regions of Asia (India, China and Japan) but also in Southern Europe, and is becoming an increasingly important forage crop in the Americas, Australia and North Africa [15]. Foxtail millet is a close relative of biofuel grasses such as switchgrass (*Panicum virgatum*) and napier grass (*Pennisetum purpureum*) and to pearl millet (*Pennisetum glaucum*), for which it represents a genetically amenable model. Moreover, the genome sequences of two foxtail millet varieties were recently released [15]–[17].

The PHT1 family of phosphate transporters, first identified and characterised in *Arabidopsis*, play critical roles in the uptake and disposition of phosphate in plants [18]. Subsequently, family members have been characterized in many plants including potato (*Solanum tuberosum*), white lupin (*Lupinus albus*), tomato (*Solanum lycopersicum*), Madagascar periwinkle (*Catharanthus roseus*), barrel medic (*Medicago trunculata*), barley (*Hordeum vulgare*), tobacco (*Nicotiana tabacum*), rice (*Oryza sativa*), maize (*Zea mays*), wheat (*Triticum aestivum*) [19] and soybean (*Glycine max*) [20].

PHT1 proteins transport Pi into cells such as the epidermal cortical cells of the root via a proton-Pi co-transport mechanism [19]. The properties of these transporters have been studied in several expression systems, including by complementation of the yeast *pho84* mutant, which lacks a high affinity phosphate transporter. The plant PHT1 proteins show different levels of affinity from high (µM range) to low (mM range) when expressed in plant cells, yeast or *Xenopus laevis* oocytes [21]–[23]. The different reported affinities and expression patterns probably reflect different functional roles such as uptake from the soil as opposed to translocation and/or remobilisation of stored Pi within the plant [19].

The arbuscular mycorrhizal (AM) symbiosis between soil fungi in the phylum Glomeromycota and the roots of c. two-thirds of all land plant species [24] is a classic mutualism whereby both partners benefit. Arbuscular mycorrhizal fungi (AMF) enhance nutrient acquisition for their host plant, particularly of poorly mobile Pi forms, through exploring a larger soil volume by extending their hyphae out into the soil and beyond the P depletion zone that builds up around the root surface [25], [26]. In return, the AMF receive a supply of photosynthetically fixed carbon from their host plant [27], which is essential for the fungus to complete its lifecycle given these fungi are obligate biotrophs. Previous work has shown that there are mycorrhiza-inducible genes encoding phosphate transporters in plants [28]. These include *MtPHT1;4* in barrel medic [29], *OsPHT1;11* and *OsPHT1;13* in rice [30], [31], *StPHT1;3*, *StPHT1;4* and *StPHT1;5* in potato [32], [33], *GmPHT1;7*,*GmPHT1;10* and *GmPHT1;11* in soybean [20], *AsPHT1;1* in *Astragalus sinicus* [28], *ZmPHT1;6* in maize [25], [34], *SlPHT1;3*, *SlPHT1;4* and *SlPHT1;5* in tomato [35], [36], and *BdPHT1;3*, *BdPHT1;7*, *BdPHT1;12* and *BdPHT1;13* in purple false brome (*Brachypodium distachyon*) [37].

However, while phosphate is known to be a major determinant of plant growth, the current literature contains no information on the effects of Pi on *S. italica* despite the fact that millets are well known for their ability to grow in unimproved soils [38]. Therefore, in the present study we examined the response of foxtail millet plants to different phosphate concentrations and demonstrated that Pi availability has a major influence on both the growth and yield of foxtail millet. To understand the underlying mechanisms by which this species responds to Pi availability, the expression patterns under the influence of different phosphate regimes have been characterised for the twelve *PHT1* family members encoded by the foxtail millet genome. The resultant information should, in the longer term, enable the development of strains of this key food crop with improved phosphate use efficiency.

## Materials and Methods

### Plant growth experiments

Seeds of *Setaria italica* cultivar ‘Maxima’ (Acc.No: Bs 3875) were obtained from the Welsh Plant Breeding Station, Genetic Resources Unit, Institute of Grassland and Environmental Research, Aberystwyth, UK and propagated by single seed descent. Plants were grown in 1∶1 (v/v) perlite:vermiculite and supplied with basal nutrient solution consisting of: 2.0 mM Ca(NO_3_)_2_, 0.5 mM MgSO_4_, 0.1 mM KCl, 10 µM H_3_BO_3_, 0.5 µM MnCl_2_, 0.5 µM ZnCl_2_, 0.2 µM CuCl_2_, 0.1 µM Na_2_MoO_4_ and 0.1 mM Fe-EDTA. The Pi concentration was varied by supplying KH_2_PO_4_ while K_2_SO_4_ was used to maintain a constant concentration of potassium in nutrient solutions of differing phosphate concentration. The plants were grown in a glasshouse with 16 h light at 26°C. Plant height, date of flowering and seed weight were recorded. Chlorophyll was measured according to [39].

For RNA isolation, seeds were sown and grown as above and supplied with nutrient solution containing either sufficient (300 µM) or deficient (10 µM) Pi. Fifteen plants were maintained at each Pi concentration. After 15 days, root and shoot portions were separated and 3 plants from each group were immediately frozen in liquid nitrogen for RNA isolation. The remaining plants were analysed for fresh and dry weights.

### Hydroponic culture

Plants, 6 per container, were grown in 10 L of nutrient solution as above but containing 300 µM or 10 µM Pi. All nutrients were prepared and diluted with de-ionized water and the pH adjusted to 6.0. Fifteen-day old seedlings grown on the inert 1∶1 (v/v) perlite:vermiculite medium and supplied with de-ionized water were transferred to hydroponic culture to initiate experiments. The pH of the nutrient solutions was checked every alternate day and adjusted to 6.0 with 0.1 M H_2_SO_4_ or 0.1 M KOH. The nutrient solutions were replaced weekly.

### Arbuscular mycorrhiza experiments

Pots (3 L) were filled with a sand and Agsorb (a calcinated attapulgite clay soil conditioner; Oil-Dri, USA) mix (1∶1, v/v) and 0.25 g L^−1^ of bonemeal (Vitax, Leicestershire, UK), a complex N and P source to encourage AM development. Mycorrhizal treatments received 100 g fresh weight inoculum of the AM fungus, *Funneliformis mosseae* (previously *Glomus mosseae*; for current phylogenetic classification see [40]) which included chopped roots, spores and growth medium. The AMF inoculum was originally obtained from Plant Works Ltd. (Kent, UK) and kept as AM pot cultures for 5 months in a glasshouse with *Plantago lanceolata* L. as the host plant in order to generate sufficient material for the subsequent experiments. The non-AM controls received the same amount of inoculum, but which had been autoclaved prior to addition [41]. To equalise the starting microbial community among the pots, 10 mL of filtered washings of the AMF inoculum, passed through a 20 µm mesh and No. 42 Whatman filter paper (Whatman International Ltd, Maidstone, UK) to remove AMF propagules, was added to the non-AM pots [42], [43]. Three seeds were sown in each pot and after germination thinned to 1 plant per pot. The plants were fed with nutrient solution as outlined above containing 30 µM Pi. Ten pots (replicates) were maintained for each AMF and non-AMF treatment. Root and leaf samples were harvested from 5 plants in each group 2 months after inoculation with the AM fungus, and the remaining plants (5 each) were allowed to grow to maturity for yield determination. For AMF root colonisation assessment, roots were cleared in 10% KOH, acidified in 1% HCl and stained with acid fuchsin (as [44] but without phenol). AMF colonisation was examined with a Nikon Optiphot-2 microscope using both brightfield and epifluorescence and x200 magnification and % root length colonisation (RLC), arbuscule and vesicle frequency recorded. At least 100 root intersections were observed for each sample [45].

### Assay of P content

Total and inorganic P contents were assayed using the modified Ames [46] protocol described by Chiou *et al*. [47].

### Semi quantitative and quantitative real-time RT-PCR

Primers were designed for all 12 SiPHT1 genes, the *actin-2* gene (*Siactin-2*) (Si026509 m.g) and the *Elongation Factor-Iα* (*EF-Iα*) gene (Si022040 m.g) using primer3 [48]. Eight of the *SiPHT1* genes do not contain introns, while four genes (*SiPHT1;9*, *10*, *11* and *12*), the *Siactin-2* gene and the *EF-Iα* gene do contain introns. Primers were designed in such a way to span the introns for all the intron-containing genes. Details of these primers, the sizes of the PCR products and the annealing temperatures (T_m_) are given in Table S1.Total RNA was isolated from leaf, root and shoot (defined as all aerial parts including leaf) using an RNeasy Plant Mini Kit (Qiagen, Manchester, UK), was treated with DNaseI (Qiagen, Manchester, UK) and then the integrity of the RNA was analyzed using an Agilent bioanalyzer (Agilent Technologies, Berkshire, UK) and quantified using a Nanodrop ND-1000 Spectrophotometer (Thermo Scientific, Wilmington, DE, USA). cDNA was made from 500 ng of RNA using a SuperScript II reverse transcriptase first-strand synthesis system (Invitrogen, Paisley, UK). Semi quantitative RT-PCR was performed in a Techne TC-512 thermal cycler (Bibby Scientific Ltd., Staffordshire, UK) using the following conditions; initial denaturation at 94°C for 2 min followed by 35 cycles of 30 s denaturation at 94°C, 30 s annealing at 58 to 62°C, based on the T_m_ of gene-specific primers (Table S1), and 30 s extension at 72°C with the final extension at 72°C for 5 min. The products were separated on 10% polyacrylamide gels. The identity of all PCR products was verified by sequencing.

For quantitative real-time PCR (qPCR), expression levels of *SiPHT1;2*, *SiPHT1;3* and *SiPHT1;4* were analysed in leaf and root samples from 15-day old hydroponically grown plants. In the mycorrhiza experiment, the expression levels of *SiPHT1;8*, *SiPHT1;9* and *SiPHT1;11* were similarly analysed. For quantification 20 µL reactions were employed containing 10 µL 2x SsoFast EvaGreen Supermix (Bio-Rad Laboratories Ltd, Hertfordshire, UK), 5 µL primers (500 nM each primer) and 5 µL diluted cDNA (1∶50). Standard curves were constructed from an appropriate range of dilutions of cDNA. The cycling conditions for qPCR were: enzyme activation at 95°C for 30 s, denaturation at 95°C for 5 s, annealing/extension at 60°C for 5 s (45 cycles), melting curve 65 to 95°C (5 s/step). The Ct (cycle threshold) values and starting quantity were calculated with CFX Manager 2.0 software. Two different control genes, *Siactin-2* (Si026509 m.g) and *EF-Iα* (Si022040 m.g) were tested for stability of expression using a range of tissues obtained from plants grown in several different conditions. Based on the Ct values, *EF-Iα* was chosen for the normalization of the expression levels in all samples. The details of these primers are given in Table S1.

### Bioinformatic analyses

The determination of the genome sequence of *S. italica* [15], [17] allowed the identification of likely orthologues of genes known to be involved in phosphate responses in other plants. To this end BLASTP searches were made of the predicted proteins encoded by the *S. italica* inbred Yugu1 genome (JGI 8.3X chromosome-scale assembly release 2.0, annotation version 2.1) at the Phytozome website (http://www.phytozome.net/) [49]. For analysis of the phylogenetic relationships between PHT1 proteins, their amino acid sequences were aligned and then the evolutionary history deduced using the Maximum Likelihood method, based on the JTT matrix-based model [50]. Evolutionary analyses were conducted using MEGA5 [51]. For analysis of the promoters of the *SiPHT1* genes, a 3000 bp region upstream of the translation start ATG was selected for each of the 12 *SiPHT1* genes identified in the *S. italica* inbred Yugu1 genome [15]. For comparison, the upstream regions of a number of mycorrhiza-inducible transporters from other cereals were also selected for such analysis. Analyses of these putative promoter regions for known *cis*-regulatory elements, including the phosphate starvation related regulatory element P1BS [52] and CTTC motif [53], [54] were performed using the PLACE database [55] or manually. In addition the EARS (Evolutionary Analysis of Regulatory Sequences) method was employed to search for evolutionarily conserved regions in the putative promoter regions of the AMF-inducible *S. italica PHT1* genes [56].

### Statistical analysis

The experiments were conducted using a randomized design. All results were analysed on SPSS 16.0 (SPSS Inc., Chicago, IL, USA) using a *t*-test at the 1% level or an one-way analysis of variance (ANOVA) with Levene’s test to assess equality of variance and a Bonferroni post-hoc test to determine significant differences among means. The number of replicates for each experiment is indicated in the figure legends.

## Results

### Phosphate influences the growth and yield of foxtail millet in glasshouse conditions

**Figure 1:**
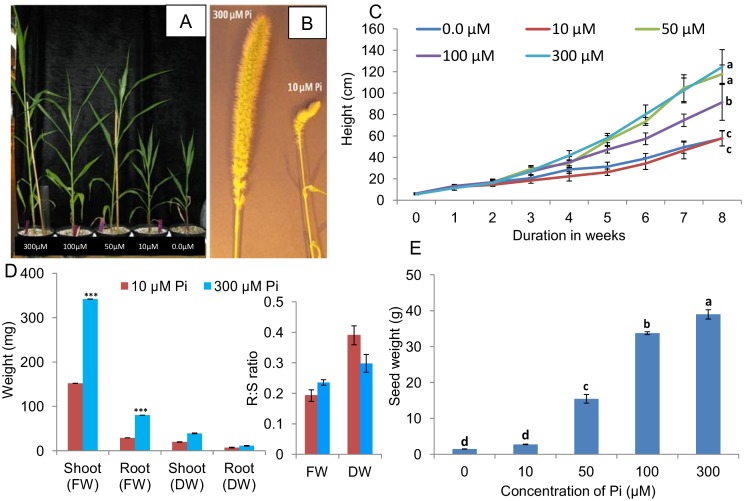
Plant growth experiments. Foxtail millet plants grown in pots containing a 1∶1 (v/v) ratio of perlite:vermiculite and supplied with nutrient solution containing various concentrations of inorganic phosphate (Pi). A, 6-week old plants grown in various concentrations of Pi; from left to right: 300 µM, 100 µM, 50 µM, 10 µM and no added Pi (0 µM); B, image of flowers representative of plants grown for 12 weeks in the presence of sufficient (300 µM) or deficient (10 µM) Pi; C, plant height measured weekly for plants grown in the presence of various concentrations of Pi. Statistical analysis was conducted at the end of the recording period (i.e. at 8 weeks); D, shoot (S) and root (R) weight (mg) and root:shoot (R:S) weight ratio of foxtail millet seedlings grown for 16 days in the presence of sufficient (300 µM) or deficient (10 µM) Pi; and E, seed yield (seed dry weight) of plants grown in the presence of various concentrations of Pi after 16 weeks of growth. Data shown are means ± standard deviation (SD), *n* = 5. Values followed by the same letter were not significantly (*P*<0.05) different based on a Bonferroni post-hoc test. For Fig. 1D, data were tested by a *t*-test; *** represents a significant difference (*P<*0.001) between the shoots or roots of plants grown with high (300 µM) compared to low (10 µM) Pi concentrations.

Plants grown at the higher concentrations of Pi were observed to be taller (Figure 1A) and produce larger full seed heads compared to those grown in low (10 µM) Pi (Figure 1B). Differences in plant height among Pi treatments could be observed within 3–4 weeks of growth (Figure 1C), and significant differences in fresh and dry weight and root to shoot ratios were apparent as early as 16 days (Figure 1D). After 5 weeks plants grown in 10 µM Pi showed the characteristic dark green appearance of phosphate-deficient plants, which was reflected in higher levels of both chlorophyll *a* and *b* (Table S2). After 8 weeks, plants grown in the absence of added Pi or with only 10 µM Pi were half the height of plants grown with 50 or 300 µM Pi and this difference was statistically significant (Figure 1C). Although the heights of plants grown in 50 µM Pi were greater than those grown on 100 µM Pi and similar to those of plants grown in 300 µM Pi, 50 µM Pi was not sufficient for optimal growth of foxtail millet as these plants had high chlorophyll content (Table S2) which is an indication of Pi stress. Seed weight produced per plant increased with increasing Pi supply, and plants grown in 50 µM produced less than half the weight of seed per plant compared to plants that received 300 µM (Figure 1E) despite there being no difference in plant height between these two Pi treatments. Plants grown in the absence of added Pi or with only 10 µM Pi took longer, on average, to flower (>85 days) compared to plants grown on 50, 100 or 300 µM Pi (ca 75 days). Based on these data, 10 µM Pi was chosen to represent Pi-deficient conditions and 300 µM Pi was used to represent Pi-sufficient conditions in subsequent experiments.

### Changes in total and inorganic phosphate content of hydroponically grown plants as a function of phosphate level in the medium

**Figure 2:**
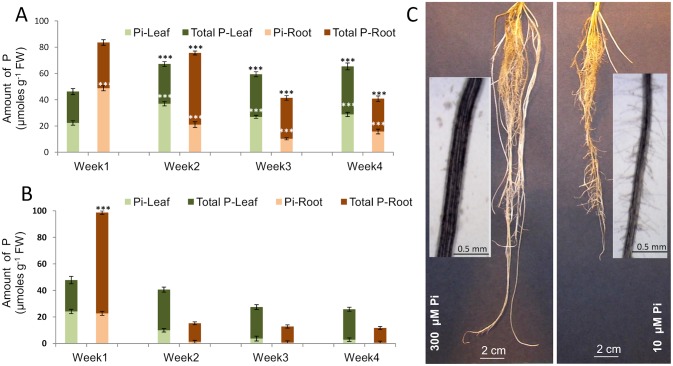
Assay of total and inorganic phosphate content in leaf and root samples. A and B, Total P and inorganic P (Pi) content in leaf and root samples of foxtail millet grown hydroponically in media containing 300 µM (A) and 10 µM (B) Pi. The total height for the bar represents total P, while inorganic P is shown within the total P bar and indicated by the lighter shading. Values shown are the means ± SD (*n* = 5). Data were analysed by a *t*-test where *** represents a significant difference (*P<*0.001) between the plants grown with high (300 µM) compared to low (10 µM) Pi concentrations. C, Root architecture of 20-day old foxtail millet plants grown hydroponically in medium containing 300 or 10 µM Pi. The insets show roots magnified to illustrate the induction of root hairs in plants grown in 10 µM Pi.

The total and inorganic P contents differed between plants grown hydroponically in high and low Pi conditions (300 µM, Figure 2A or 10 µM, Figure 2B). After 1 week, plants grown in both concentrations showed a higher level of total P in the roots than in the leaves (Figure 2A and B). There was no significant difference between the amount of total P in leaf samples from the plants grown in 300 µM (Figure 2A) and 10 µM (Figure 2B) Pi in the first week of the experiment. In contrast, the amount of Pi was significantly higher in the roots of the plants grown in 300 µM Pi and total P concentration was significantly higher in the plants grown on 10 µM Pi (Figure 2B). In the plants grown in Pi-sufficient conditions (Figure 2A), the levels of both total P and Pi in the roots declined between week 1 and 3 then stabilised. In contrast, leaf total P and Pi increased after week 1, and Pi was between 40–50% of total leaf P. In the plants grown in Pi-deficient conditions (Figure 2B) both the total and inorganic P levels declined very rapidly in the roots and more gradually in the leaf tissue between week 1 and week 2, and were significantly lower compared to plants grown in higher concentrations of Pi. Overall, Pi amounts were virtually undetectable in the roots and maintained at a very low level in the leaf tissue of the plants grown in 10 µM Pi after the first week.

The plants grown in Pi-deficient conditions (10 µM) produced shorter, denser roots with more root hairs compared to those grown under Pi-sufficient conditions (300 µM) (Figure 2C). The latter plants also exhibited fewer lateral roots.

### The *PHT1* phosphate transporters of foxtail millet; comparison with those of other plants

**Figure 3:**
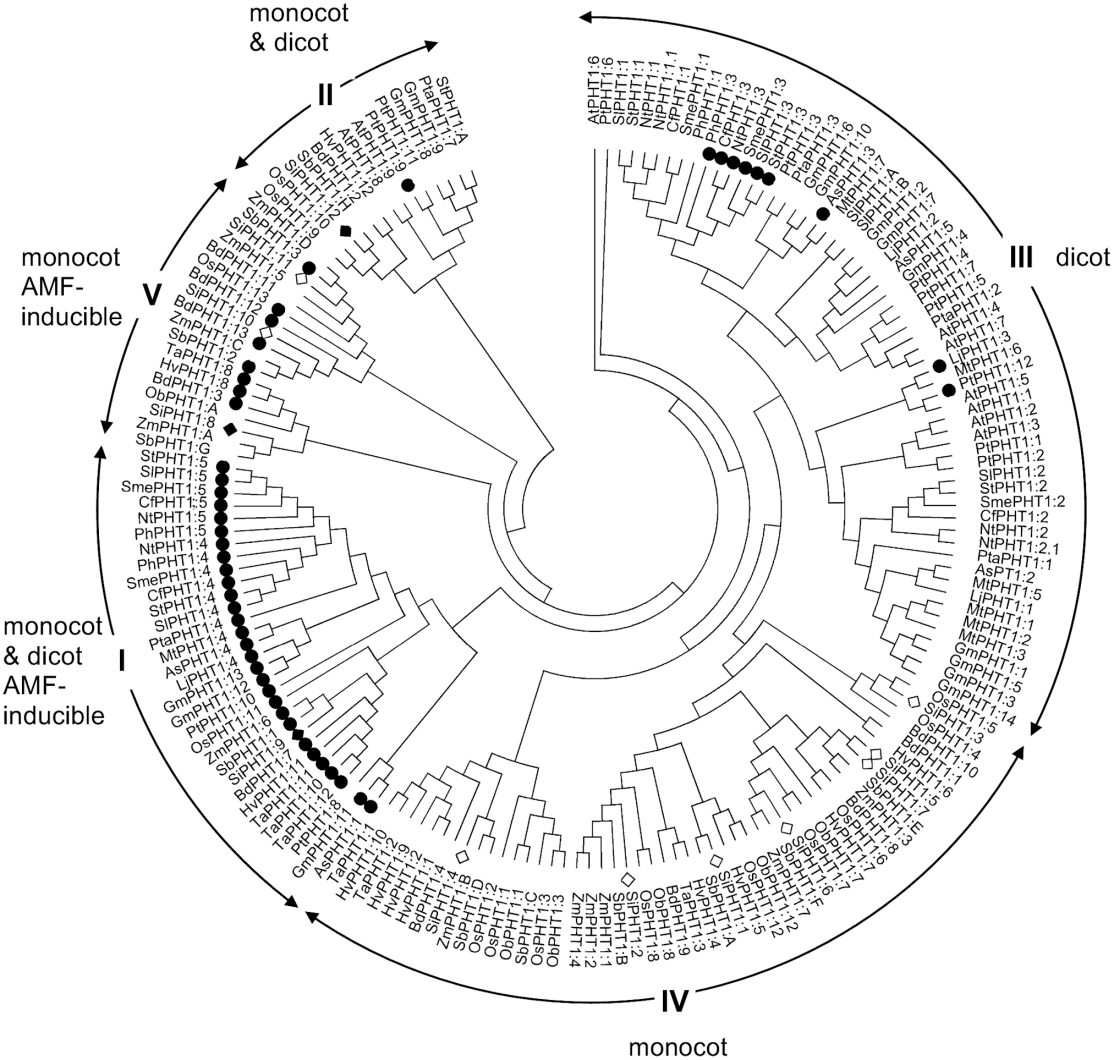
Phylogenetic analysis of plant *PHT1* family members. Roman numerals (I–IV) indicate the four *PHT1* subfamilies identified by Nagy *et al*. [33] together with a more-recently identified family of arbuscular mycorrhizal fungus (AMF)-inducible transporters (V) specific to the Poaceae [65]. Sequence names start with the first letter of the genus and the first one or two letters of the species name, followed by the gene name. Accession numbers for the proteins are given in Table S5. *PHT1* family members from *S. italica* are indicated by open diamonds or, in the case of AMF-inducible members, filled diamonds. Other plant *PHT1* family members that have been described to be AMF-inducible are indicated by filled circles.

Genes encoding twelve *PHT1* family phosphate transporters (Table S3) and two apparent pseudogenes (Gene loci Si013484 m.g and Si035855 m.g) were identified in the *S. italica* genome. Phylogenetic analysis of the predicted PHT1 protein sequences revealed that these could be clustered with orthologues previously identified in other members of the Poaceae, in particular purple false brome (*Brachypodium distachyon*), rice, sorghum (*Sorghum bicolor*) and maize (Figure 3).

### Expression analysis of 12 *SiPHT1*s in leaf and root samples of hydroponically grown plants by semi quantitative RT-PCR

**Figure 4:**
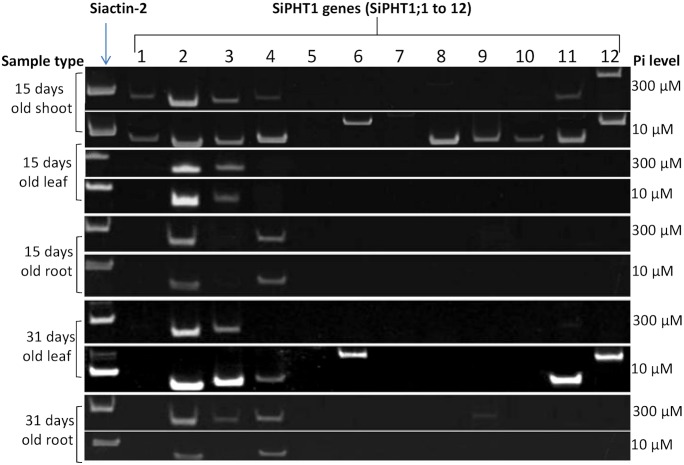
RT-PCR analysis of expression patterns of the foxtail millet *PHT1* gene family. cDNA produced by reverse transcription of mRNA was prepared from various tissues of plants grown in Pi**-**deficient (10 µM) and Pi-sufficient (300 µM) conditions and then amplified with primers specific for each of the 12 *SiPHT1* genes and for the *Siactin-*2 gene. PCR products were separated on 10% polyacrylamide gels and visualized using SYBR safe DNA gel stain. The 15 and 31 d leaf and root samples were obtained from hydroponically grown plants; 15 dshoot was obtained from pot grown plants (perlite:vermiculite).

To gain an overview of the expression of the PHT1 family RT-PCR was performed for the twelve *SiPHT1* genes in the ‘shoot’ (all aerial tissues including leaves), leaf and root (Figure 4). The shoot tissue of 15-day old seedlings showed the expression of many members of the *PHT1* gene family. The transcripts of six genes, *SiPHT1;1*, *SiPHT1;2*, *SiPHT1;3*, *SiPHT1;4*, *SiPHT1;11* and *SiPHT1;12*, were detected both in plants grown in Pi-sufficient conditions (300 µM Pi) and in plants grown in Pi-deficient conditions (10 µM Pi). In contrast the transcripts of four genes, *SiPHT1;6*, *SiPHT1;8*, *SiPHT1;9* and, *SiPHT1;10*, were only detectablein shoot samples from plants grown under Pi-deficient conditions (10 µM Pi), indicating that transcription of these genes was strongly induced by P-starvation.

In 15-day old leaf *SiPHT1;2* and *SiPHT1;3* were the main transporters detected, whereas in 15-day root *SiPHT1;2* and *SiPHT1;4* were the principal transporters expressed. In older (31-day) leaves, expression of 4 additional transporters, *SiPHT1;4*, *SiPHT1;6*, *SiPHT1;11* and *SiPHT1;12*, was induced by growth in Pi-deficient conditions (10 µM Pi). In 31-day old root tissue no additional transporters appeared to be up-regulated by growth in Pi-deficient conditions (10 µM Pi) but two additional transporters, *SiPHT1;3* and *SiPHT1;9*, were detectable in plants grown in Pi-sufficient conditions.

### Expression analysis of *SiPHT1;2*, *SiPHT1;3* and *SiPHT1;4* in leaf and root samples of hydroponically grown plants by quantitative real time RT-PCR

**Figure 5:**
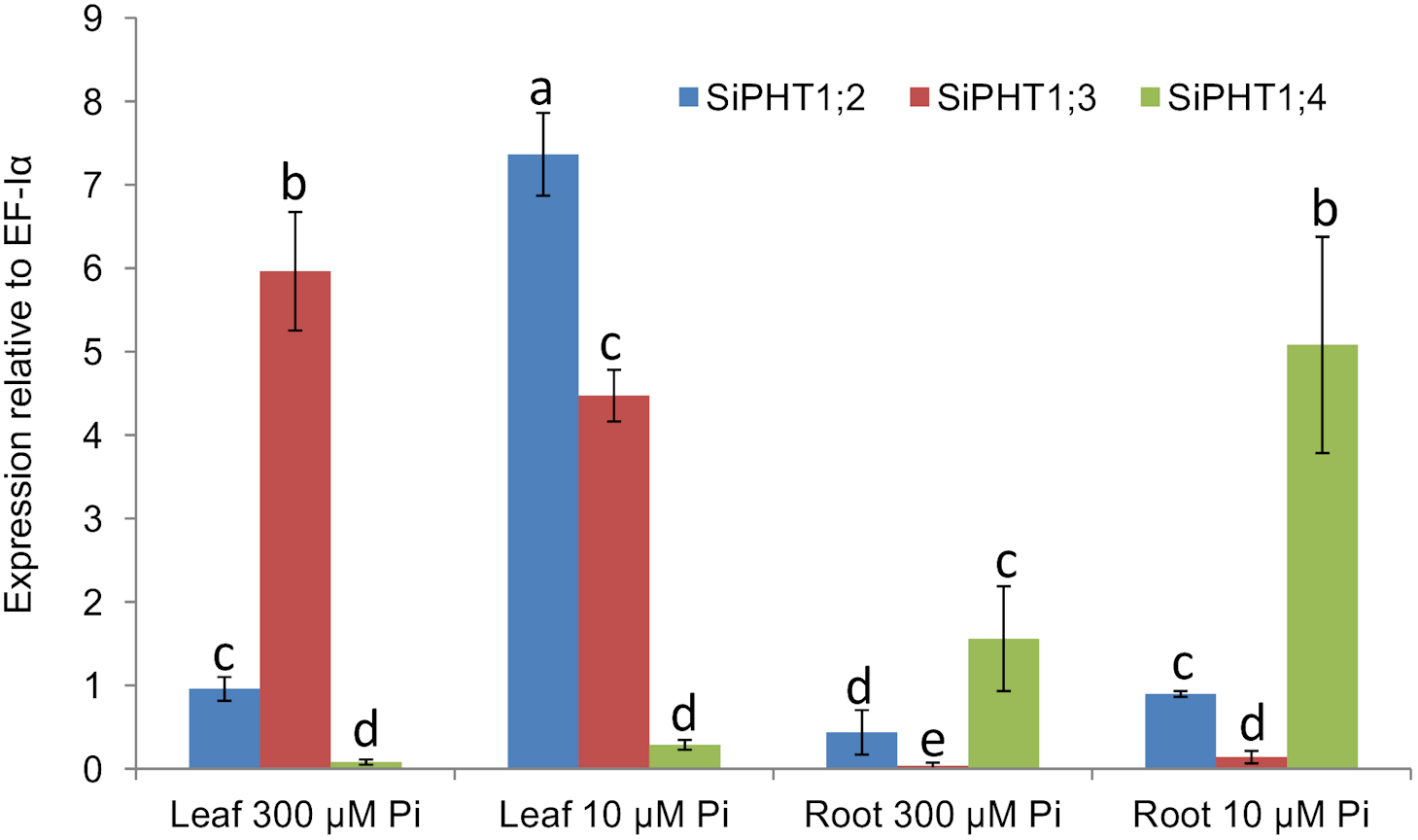
Quantitative real-time PCR analysis of *SiPHT1*;2, *SiPHT1*;3 and *SiPHT1*;4. Quantitative real-time PCR analysis of *SiPHT1*;2, *SiPHT1*;3 and *SiPHT1*;*4* expression in leaf and root samples of 15 d foxtail millet plants grown hydroponically in media containing either 300 µM or 10 µM Pi. Values are mean ± SE of 3 biological replicates each consisting of 3 technical replicates. The values were compared by one way ANOVA for the expression of genes. Values indicated by the same letter are not significantly different (*p*<0.05), based on a Bonferroni post-hoc test for the expression level of the same gene in different tissues.

In order to obtain more quantitative information on expression levels in response to Pi supply, quantitative real time PCR was carried out for the *SiPHT1;2*, *SiPHT1;3* and *SiPHT1;4* genes using cDNA from 15-day old hydroponically grown root and leaf samples. Based on the Ct values *EF-Iα* was chosen as a reference gene for the normalization of *SiPHT1* isoform expression levels as compared to *Siactin-2*, *EF-Iα* showed less variation in Ct values among different samples (Table S4). This finding is in agreement with a recent study that reported that *EF-Iα* is superior to *Siactin-2* as a reference gene in foxtail millet [57]. As indicated above, the results of RT-PCR analysis suggested that *SiPHT1;2, SiPHT1;3* and *SiPHT1;4* are the most widely expressed transporters in millet (Figure 4).The results confirmed that *SiPHT1;2* is expressed in both roots and leaves of plants grown under Pi-deficient and Pi-sufficient conditions. Expression levels were significantly higher in leaves of P-deficient plants, where expression was strongly (>7 fold) induced by growth in Pi-deficient conditions (Figure 5). Expression of this transporter in the root sample was also up-regulated by low Pi and significantly higher levels of expression were seen, but to a lesser degree than in the leaf. The expression of *SiPHT1;3* predominantly in leaves was also further substantiated by qPCR, as was a slight decrease in expression levels in samples from plants grown in Pi-sufficient conditions. Similarly, the results of qPCR confirmed that *SiPHT1;4* is expressed predominantly in roots, with approximately 3-fold higher expression in samples from plants grown in Pi-deficient conditions (10 µM Pi) than in samples from plants grown in Pi-sufficient conditions (300 µM Pi). Such induction of expression by growth in Pi-deficient conditions had not been apparent from simple RT-PCR analysis (Figure 4).

### Expression analysis of *SiPHT1* family members in response to colonisation by *F. mosseae*

**Figure 6:**
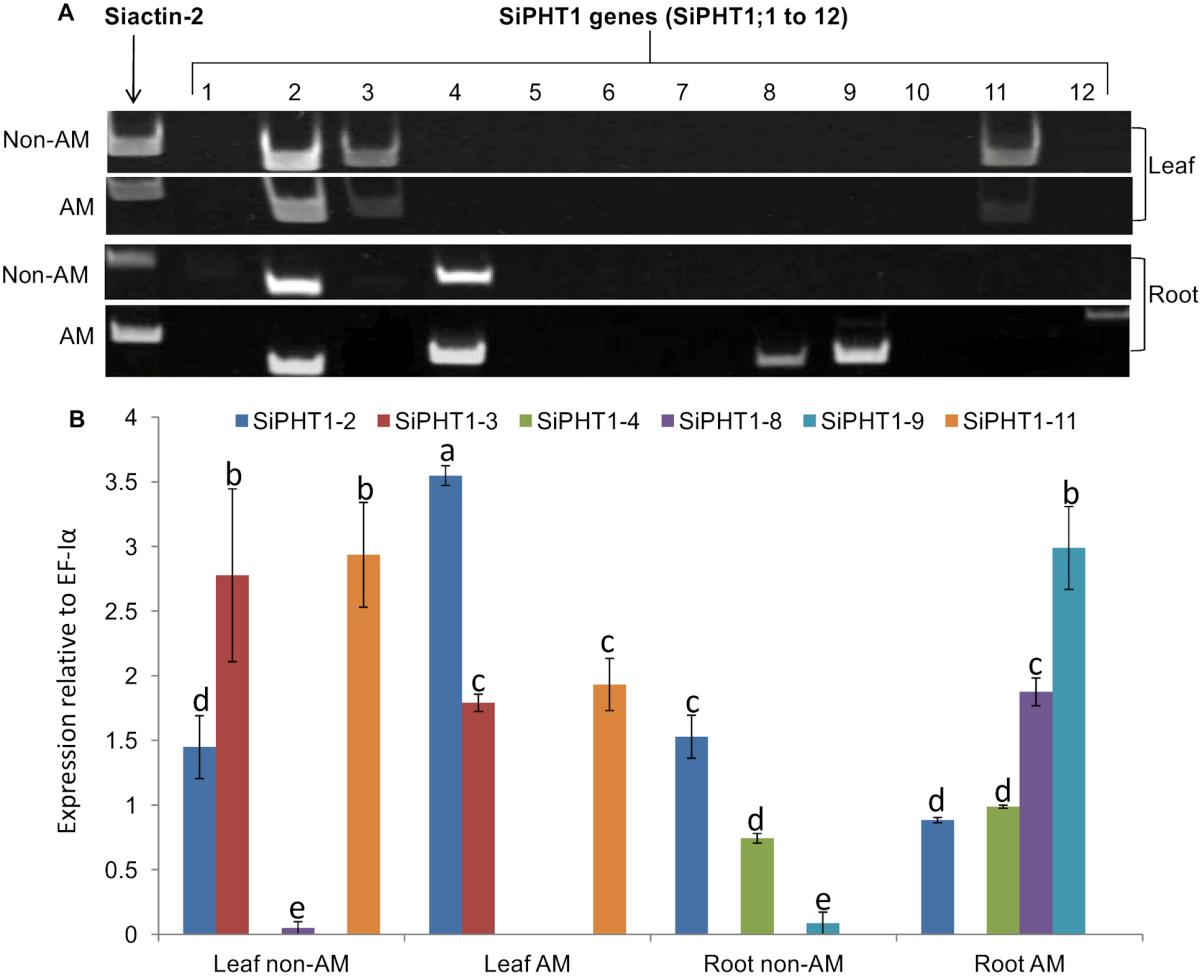
Expression analysis of *SiPHT1* genes in roots and leaves of plants colonised by *F. mosseae*. A; expression analysis by semi quantitative RT-PCR. cDNA was prepared and PCR performed as described in the legend to Figure 4. B; expression analysis by quantitative real-time PCR of *SiPHT1;2*, *SiPHT1;3*, *SiPHT1;4*, *SiPHT1;8*, *SiPHT1;9* and *SiPHT1;11* in leaf and root samples of 2 month old foxtail millet AM or non-AM plants. Values are mean ± SE of 3 biological replicates each consisting of 3 technical replicates. The values were compared by one way ANOVA for the expression of genes. Values indicated by the same letter are not significantly different (*p*<0.05), based on a Bonferroni post-hoc test for the expression level of the same gene in different tissues.

*S. italica* plants were grown with live (AMF) or autoclaved AMF inoculum (non-AMF) of *F. mosseae* as described in the Methods section. After 2 months RNA was extracted from leaf and root samples for RT-PCR and root samples assessed for AMF colonisation. The AM roots showed colonisation of between 17 and 29% (23±2.5%) of the root length. Arbuscules were less frequent (0.75±0.5%), while vesicles were absent. Extraradical mycelium and attached spores were also observed outside the root, while no colonisation by AMF was observed in roots from the non-AMF treatment. In addition to bands for *SiPHT1;2* and *SiPHT1;4*, which had been previously found in non-AM colonised plants (Figure 4), RT-PCR analysis of root samples from AM colonised plants revealed clearly detectable bands for *SiPHT1;8*, and *SiPHT1;9*, indicating the induction of these 2 genes by AM fungal colonisation (Figure 6A). A lower-intensity band corresponding to *SiPHT1;12* was also evident in the same root sample. Expression of *SiPHT1;8*, *SiPHT1;9* and *SiPHT1;12* was not detectable in root samples from the non-AM colonised plants, confirming that expression of these genes was specifically induced by AMF colonisation. The leaf samples showed expression of *SiPHT1;11* in addition to *SiPHT1;2* and *SiPHT1;3* consistent with the results of previous experiments (Figures 4 and 5).

Based on these results six SiPHT1 isoforms (*SiPHT1;2*, *SiPHT1;3*, *SiPHT1;4*, *SiPHT1;8*, *SiPHT1;9* and *SiPHT1;11*) were selected for further analysis by qPCR. The results confirmed the specific induction of *SiPHT1;8* and *SiPHT1;9* in mycorrhizal roots as expression of these two genes was not detected in leaf samples from AM colonised plants or in non-AM leaves or roots (Figure 6B), although their expression had previously been detected by semi-quantitative RT-PCR in shoots of un-colonised plants (Figure 4). Of these two genes induced by mycorrhiza in roots, *SiPHT1;9* showed significantly higher expression compared to *SiPHT1;8*. *SiPHT1;11* was expressed in leaf samples but was not specific for mycorrhizal plants, indeed the level of expression was higher in the non-AM control plants than in the AM colonised plants. Moreover, as found in previous experiments, *SiPHT1;2* was expressed ubiquitously.

### Arbuscular mycorrhizal colonisation improves the seed yield of foxtail millet

**Figure 7:**
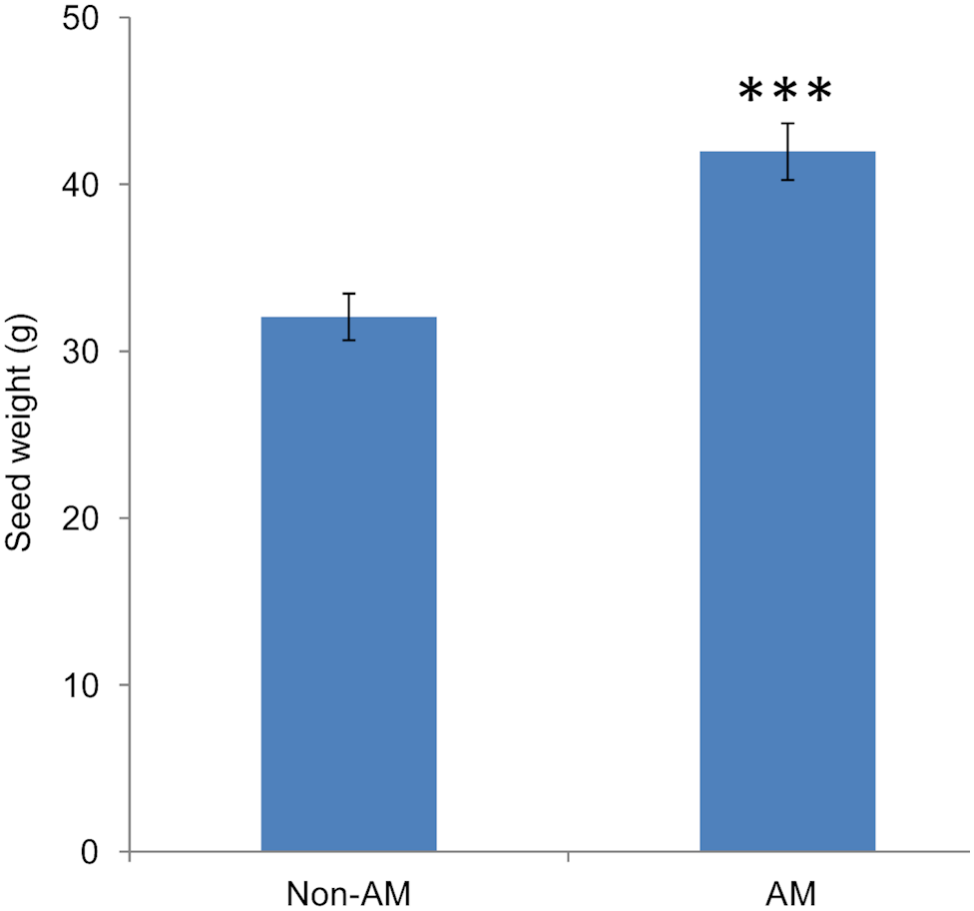
Seed yield (dry weight) of AM or non-AM foxtail millet. The seeds were harvested after 16 weeks of growth. Values are mean ± SD (*n* = 5). Data were tested using a t-test where *** = *P*<0.001.

The AM plants produced a significantly higher seed yield than the non-colonised control plants (Figure 7). This result is consistent with the main role of AMF in capturing poorly mobile phosphate ions from the soil environment and transferring this phosphate to their associated host plant.

### Bioinformatic analysis of promoters

**Figure 8:**
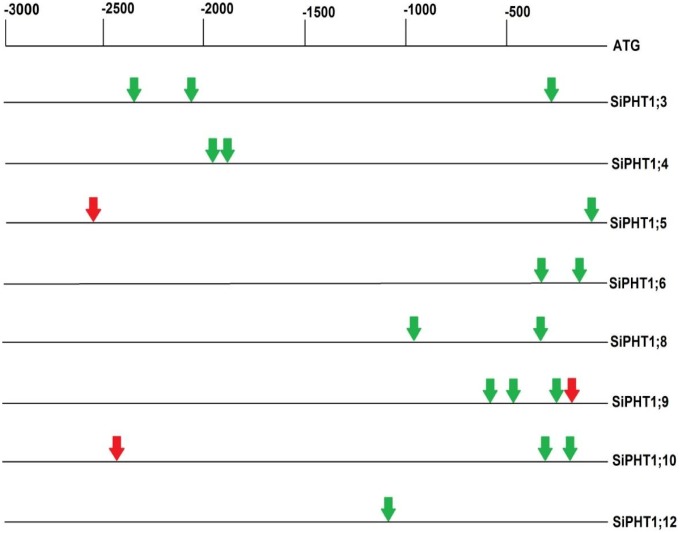
Schematic diagram showing the locations of the P1BS and CTTC motifs in promoter regions of 8 *SiPHT1s*. The P1BS and CTTC motifs are shown in green and red respectively and are located between −1 to −3000 bp upstream of the start codon ATG.

Bioinformatic analysis revealed the presence of several types of known cis-regulatory elements in the putative promoter regions of the *SiPHT1* genes. In particular, P1BS motifs were found in the promoter regions of *SiPHT1;3*, *SiPHT1;4*, *SiPHT1;5*, *SiPHT1;6*, *SiPHT1;8*, *SiPHT1;9*, *SiPHT1;10* and *SiPHT1;12* (Figure 8). The P1BS motif is an 8 bp sequence (GNATATNC) present in many phosphate starvation responsive genes in plants [52]. A second motif, the “CTTC” motif [53], comprising a 7 bp core sequence (TCT(T/C)GTT) previously identified as being present in the promoters of multiple AMF-inducible *PHT1* genes in both subfamilies I and V [54], was found in the putative promoter regions not only of the AMF-inducible *SiPHT1;9* gene but also in the promoters of *SiPHT1;5* and *SiPHT1;10*, which were not found to be induced by AMF in the current study. Investigation of the promoter regions of *SiPHT1;9* and related family I AMF-inducible genes from other monocots (*SbPHT1;1*, *ZmPHT1;6*, *BdPHT1;7* and *OsPHT1;11*) using the EAR algorithm [56] revealed that the CTTC motif was present, together with an upstream P1BS motif, in a conserved region corresponding to −263 to −173 of the *SiPHT1;9* promoter (Figure S1).

## Discussion

### Phosphate influences the growth and yield of foxtail millet in glasshouse conditions

Before embarking upon characterisation of the expression pattern of the *SiPHT1* family genes in response to Pi, it was necessary first to characterise growth of *S. italica* under conditions of varying P supply. Plant height, fresh and dry weights, time to flowering, seed yield and determination of total and inorganic phosphate content were measured and based on these data 10 µM Pi was selected as representative of phosphate-deficient conditions whilst 300 µM Pi was taken as being phosphate-sufficient for subsequent experiments. The latter concentration is similar to that required by rice [58], while 100 µM Pi is sufficient for optimal growth of barley [59].

The root morphology of plants grown hydroponically with 10 µM Pi showed shortened primary root, increased root hairs and lateral roots. This type of change in root architecture induced by P starvation has been well documented in many species including *Arabidopsis* [60], [61] and Lupin [62], [63]. The proliferation of root hairs under P starvation is believed to increase the adsorptive surface, to increase exudation of organic acids and phosphatases, and possibly to result in greater expression of Pi transporters [64].

### *PHT1* transporter gene family of foxtail millet

The *PHT1* gene family of phosphate transporters in plants comprises multiple members which can be assigned to one of 5 subfamilies based on sequence similarity [33], [65]. They include both low and high affinity transporters and whilst most family members are expressed in roots, individual members have complex and overlapping expression patterns reflecting different physiological functions [19], [66]. For example *OsPHT1;6* is a high affinity transporter with an apparent *K*_m_ of 97 µM when expressed in yeast, and is expressed in root epidermal cells where it is likely responsible for Pi uptake from the soil. In contrast *OsPHT1;2* is expressed throughout the stele, exhibits an apparent *K*_m_ in the mM range when expressed in *Xenopus* oocytes, and may be involved in translocation of stored Pi in the plant [21].

The foxtail millet *PHT1* family comprises 12 members (Figure 3) and evidence for expression of all genes except the closely related *SiPHT1;5* and *SiPHT1;7* was obtained (Figure 4). *SiPHT1;2* was found to be expressed in all the tissues and conditions tested in this study, and would appear to be a widely expressed *PHT1* transporter with higher expression in leaf (and to a lesser extent root samples) of Pi-depleted plants (Figure 5). *SiPHT1;2* grouped in the phylogenetic tree with *OsPHT1;8* (Figure 3) which has been shown to be a high affinity transporter expressed under all conditions although, unlike *SiPHT1;2*, *OsPHT1;8* is more highly expressed in roots than shoots [23]. *OsPHT1;8* is only 1.5-fold up regulated by P deficiency in roots and is not affected by P supply in leaf tissue [23]. However, in another study, expression of *OsPHT1;8* was also found to be up-regulated in the shoot by P deficiency [67]. *SiPHT1;2* also grouped with *BdPHT1;9*, which displays both shoot and root expression [37], as did maize *PHT1;1* and *PHT1;4*/*PHT1;2* [37], [68]. Like *SiPHT1;2*, *ZmPHT1;2* was up regulated in both the root and leaf tissues by P deficiency [68].

In contrast to *SiPHT1;2*, expression of *SiPHT1;3* was found to be predominantly expressed in leaf tissue in 15-day old hydroponically grown plants and rather than being induced by Pi-deprivation was slightly decreased under such conditions (Figure 5). However, its expression was also detectable in the roots of plants grown for 31 days in Pi-sufficient conditions (Figure 4). Similar predominant expression of some *PHT1* family members in tissues other than roots has been reported in other plant species. For example, in Arabidopsis *AtPHT1;5* was expressed in cotyledons and hypocotyl during early seedling growth and was suggested to be involved in remobilisation of stored phosphate from phytate. At later times, expression was confined to vascular tissues and the transporter may be involved in remobilisation of phosphate from senescing leaves [69]. A transporter from purple false brome, *BdPHT1;10*, closely related to *SiPHT1;3* (Figure 3), has been shown to be expressed in shoots but not roots [37].

Conversely *SiPHT1;4* is predominantly expressed in roots and induced by low Pi (Figure 5), but is also detected in aerial tissue under low Pi conditions (Figure 4). Phylogenetic analysis revealed that *SiPHT1;4* is grouped with *BdPHT1;4* of purple false brome, which is expressed in roots but not shoots [37]. In rice and Arabidopsis, where expression of the *PHT1* family has been most extensively studied, the majority of family members are expressed in roots. *AtPHT1;1*, *AtPHT1;2*, *AtPHT1;3* and *AtPHT1;4* are expressed in the root epidermis and induced by low Pi. *AtPHT1;7*, *AtPHT 1;8* and *AtPHT1;9* are also expressed at low levels in Pi starved roots [69], while both *AtPHT1;1* and *AtPHT1;4* have been shown to play a major role in Pi acquisition in Arabidopsis [70], [71]. *OsPHT1;4* and *OsPHT1;8* were expressed in roots of 3 rice cultivars [66] and *OsPHT1;6* was shown, through RNA interference, to play an important role in Pi uptake while *OsPHT1;2* was suggested to be involved in the root to shoot transport of Pi [21]. Barley *HvPHT1;1*, which is expressed in roots, is moderately up regulated by P deficiency and encodes a high affinity P transporter [72]. It also groups with *SiPHT1;4* in the phylogenetic tree (Figure 3). We did not observe any evidence for other *SiPHT1* family members being induced by low Pi in roots but RT-PCR and qPCR analysis of bulk tissues may of course fail to detect genes that are highly expressed in very specific but quantitatively minor locations, such as root tips or root hairs. Other methods which preserve spatial information, such as use of promoter reporter fusions, will be required to explore such possibilities.

### Colonisation with the arbuscular mycorrhizal fungus *F. mosseae* induces the expression of *SiPHT1;8* and *SiPHT1;9* in roots

Arbuscular mycorrhizal fungi have previously been demonstrated to confer a wide range of benefits to their associated host [27] and plants may get 70% of their phosphate [65] via AM fungi, in addition to acquiring other limiting resources such as nitrogen [73] and water [74]. Consistent with this, the seed yield of AM plants showed a 30% increase over the non-AM control plants (Figure 7), even though both sets of plants were grown in the presence of a small quantity of bonemeal to encourage AM symbiosis establishment and therefore were not as phosphate deficient as the plants grown hydroponically with 10 µM Pi. Thus, understanding this important plant-fungal interaction and ensuring maximum benefit to the plant is an attractive means to improve yields in low input agricultural systems.

Arbuscular mycorrhizal colonisation induced the expression of *SiPHT1;8* and *SiPHT1;9* in roots, as demonstrated by semi-quantitative and quantitative RT-PCR, while *SiPHT1;2* showed a more than 2-fold increase in expression in the leaves of AM plants whereas *SiPHT1;11* expression was reduced. Weak, but detectable, selective expression of *SiPHT1;12* was also observed in AM roots (Figure 6). As mentioned in the introduction, AM-inducible *PHT1* transporters have been described in many monocot and dicot species, and their role in the symbiosis is supported by the observation that typically they are expressed exclusively in arbuscule-containing root cells [29]. However, in some cases, low levels of expression in un-colonised plants, and in tissues other than roots, has been detected [75], suggesting additional roles in phosphate homeostasis. Such additional roles for *SiPHT1;8* and *SiPHT1;9* are clearly suggested from the observation in the present study of their expression in the shoots of un-colonised plants (Figure 4). In order to probe such roles, further work will be required to localise the expression of these transporters to specific tissues. *SiPHT1;9* is a member of subfamily I of the *PHT1* transporters, an evolutionarily ancient grouping which contains many AMF-inducible transporters from both monocots and dicots (Figure 3). These include *HvPHT1;11* from barley [76] and *OsPHT1;11* from rice [30]. In contrast, *SiPHT1;8* is a member of subfamily V, a group of AMF-inducible transporters that arose relatively recently in the Poaceae [65] and which is exemplified by *HvPHT1;8* from barley [77] and *OsPHT1;13* from rice [31], [65]. In addition to playing key roles in symbiotic phosphate uptake by plants, there is evidence that AMF-inducible *PHT1* transporters also play a role in controlling the development and lifespan of arbuscules [65]. Interestingly, while both *OsPHT1;11* and *OsPHT1;13* are required for the proper development of the AM symbiosis in rice, only the former contributes to symbiotic Pi uptake [65]. Further investigations, for example using RNAi, will be required to assess whether *SiPHT1;9* and *SiPHT1;8* play corresponding roles in millet.

### Promoters of foxtail millet *PHT1* contains regulatory elements specific for the expression by Pi starvation and arbuscular mycorrhizal colonisation

Identification of P1BS motifs in the putative promoter regions of several of the *SiPHT1* genes is consistent with their induction by Pi starvation, as has been reported for *PHT1* promoters in other plant species such as rice. For example the promoter region of *SiPHT1;4*, expression of which is increased by Pi starvation in roots (Figure 5), contains two P1BS motifs.

Identification of a CTTC motif in the putative promoter region of the *SiPHT1;9* gene is consistent with the induction of expression of this gene in roots as a result of AMF colonisation. While first identified in the promoter of the AMF-inducible potato transporter *StPHT1;3* [53], this motif has subsequently been reported in the promoters of many members of the *PHT1* subfamily I in dicots, and most recently in that of subfamily V gene *OsPHT1;11* from rice [54]. Using deletion analysis of the *StPHT1;3* promoter and other approaches, this motif was demonstrated to be necessary and sufficient for the transcriptional response to AMF colonisation under low Pi conditions [54]. However, the results of analysis of the promoters of AMF-inducible *PHT1* genes from tobacco and eggplant using deletions and mutations suggest that both a CTTC motif and an upstream P1BS motif is required for high-level AMF-inducible transcription [52]. Such an arrangement of motifs is evident in the putative *SiPHT1;9* promoter as well as in the promoters of other AMF-inducible monocot *PHT1* subfamily I genes including *OsPHT1;11* from rice [54] (Figure S1).

While the promoter of the AMF-inducible subfamily V *OsPHT1;13* gene from rice also contains a CTTC motif [54], no such motif is present in the promoter of *SiPHT1;8* (Figure 8), despite its induction following AMF colonisation (Figure 5). Thus, the promoter elements responsible for the induction of expression of *SiPHT1;8* by AMF colonisation remain unclear and require experimental determination in future.

## Conclusion

In conclusion, we have characterised the growth response of foxtail millet to different levels of Pi and shown that despite this crop being typically grown in unimproved soils, optimal growth and yield requires 300 µM Pi, similar to that required by rice. Therefore development of millet plants with improved P acquisition and use efficiency could provide a significant benefit to resource poor farmers. We identified the 12 members of the PHT1 family of phosphate transporters and characterised their expression in response to Pi supply and AMF colonisation as a first step towards development of improved millet varieties by breeding or biotechnological approaches.

## Supporting Information

Figure S1 **Alignment of conserved regions in the promoters of the AM-inducible genes** ***SiPHT1;9*** **,** ***SbPHT1;1*** **,** ***ZmPHT1;6*** **,** ***BdPHT1;7*** **and** ***OsPHT1;11*** **.** Numbering shown is relative to the start codon ATG. The regions corresponding to the P1BS and CTTC motifs are highlighted in green and cyan respectively. Positions identical in all sequences are indicated with an asterisk.
(DOCX)

Table S1 **Details of primers with annealing temperatures (Tm) used for RT-PCR and qPCR experiments.**
(PDF)

Table S2 **Effect of phosphate on the pigment content of foxtail millet.**
(PDF)

Table S3 **Gene and protein details of foxtail millet** ***PHT1*** **phosphate transporters and reference genes.**
(DOCX)

Table S4 **Cycle threshold (Ct) values of** ***Siactin-2*** **and** ***EF-Iα*** **in qPCR analysis.**
(DOCX)

Table S5 **Plant** ***PHT1*** **sequences employed for phylogenetic analysis shown in** **Figure 3** **.**
(DOCX)
