# Supplementary material for: Phosphate Concentration and Arbuscular Mycorrhizal Colonisation Influence the Growth, Yield and Expression of Twelve PHT1 Family Phosphate Transporters in Foxtail Millet (Setaria italica)
Source: PLoS One. 2014 Sep 24;9(9):e108459. doi: 10.1371/journal.pone.0108459 (PMC4177549; doi:10.1371/journal.pone.0108459)
Supplement: Figure S1 — Alignment of conserved regions in the promoters of the AM-inducible genes SiPHT1;9 , SbPHT1;1 , ZmPHT1;6 , BdPHT1;7 and OsPHT1;11 . Numbering shown is relative to the start codon ATG. The regions corresponding to the P1BS and CTTC motifs are highlighted in green and cyan respectively. Positions identical in all sequences are indicated with an asterisk. (DOCX) [file pone.0108459.s001.docx]

**Figure S1**

**Alignment of conserved regions in the promoters of the AM-inducible genes *SiPHT1;9*, *SbPHT1;1*, *ZmPHT1;6*, *BdPHT1;7* and *OsPHT1;11*.**. Numbering shown is relative to the start codon ATG. The regions corresponding to the P1BS and CTTC motifs are highlighted in green and cyan respectively. Positions identical in all sequences are indicated with an asterisk.

**P1BS motif**

*SbPHT1_1* ------CGAGCACCTAGTGATCAAGCCAAGCGGAATATGCTGACAA-CCAGCACGT---- -203

*SiPHT1_9* AG--ATCGAGCA--TAGTGCTC-----GAGCGGAATATGCTGCCAA-CCAGCACGAGACA -249

*ZmPHT1_6* AG--AGTGCCTGCCTAGTGATC-----AAGCGGAATATGCTGCCAA-TCAGCACGAGACG -231

*BdPHT1_7* TGCTATTGC-TACCTAGTTCAC-----ACGTCGAATATGCTGCCAA-CCAACACGAGACA -212

*OsPHT1_11* CGC--CTAGACGCCTAGTGTAC-----GCGCCGAATATGCTGCCAAACCAACACGGGACA -218

**** * * ********** *** ** ****

**CTTC core motif**

*SbPHT1_1* -----------------------CGTCCTTGGCCTCGTTGCAGACGCCCCCCTCTCGTTC -152

*SiPHT1_9* GGGGA------CGCGGATGACGACGGGGTTCGCCTCGTTGCAGACGCCCCCCTCTCGTTC -177

*ZmPHT1_6* GGGA--------GCCAATGATGACGGCCTTGGCCTCGTTGTAGACGCCCC--TCTCGTTC -163

*BdPHT1_7* AAGACGGAGCACACGAAAGACGACAGTTTTC-CCTCGTTGCAGCCGTCC---TCTGGTTC -137

*OsPHT1_11* ATCTCC-----CGCGCTTGGCGACAGC-TTCTCCTCGGTGCAGACGCCC---TCTCGTTC -148

* ** ***** ** ** ** ** *** ****
