# Supplementary material for: Phosphate Concentration and Arbuscular Mycorrhizal Colonisation Influence the Growth, Yield and Expression of Twelve PHT1 Family Phosphate Transporters in Foxtail Millet (Setaria italica)
Source: PLoS One. 2014 Sep 24;9(9):e108459. doi: 10.1371/journal.pone.0108459 (PMC4177549; doi:10.1371/journal.pone.0108459)
Supplement: Table S1 — Details of primers with annealing temperatures (Tm) used for RT-PCR and qPCR experiments. (PDF) [file pone.0108459.s002.pdf]

Table S1

Details of primers with annealing temperatures (T_m) used for RT-PCR and qPCR experiments

Name of the gene	Forward primer (5' to 3')	Reverse primer (5' to 3')	Product length (bp)	T_m (°C)
SiPHT1;1	CTCATCCTCTCCACGGTGTT	CCGCCAGTAGTAGGTCAGGA	150	60.1
SiPHT1;2	ACCAGGACAAGAGCAAGGTG	GGCACGAGGAACGTGAGTAT	115	60.2
SiPHT1;3	TGTCATCGGGTTCTTGTTCA	AATTGGTCGGAACAGTCTGC	128	60.1
SiPHT1;4	CAGAAGGAGATCCAGGACGA	CGATATCGAGCAGGAACCAC	145	60.4
SiPHT1;5	CAAATGACGACGACCACAAA	AAGCCGTACATGACGACGAA	427	61.0
SiPHT1;6	CGATCTTCAAGAGCCAGTTCC	GCGAACTCCCAGGAGAAGAG	325	61.5
SiPHT1;7	CAAATGACGACGACCACAGG	AAGCCGTACATGACGACGAA	427	62.0
SiPHT1;8	GGATACTCTTCACCTGCTTCCT	TACAGCGGTAGAATCTGGGAGT	133	59.5
SiPHT1;9	CAAGGAGATAAACGCCCTGAC	ACCGATGAGCTGGATCAGGTA	142	61.4
SiPHT1;10	GTGGCGACTACCCACTATCAG	CGCTGAGACGACCAATACAAC	140	60.1
SiPHT1;11	GGAGTACGCCAACAAGAAGAGA	GATGTGGTGCTGTTCAGAAAGG	129	61.3
SiPHT1;12	GTCACCTTCTACTGGAGGATGG	TGTAGTAGGGGATGTCCAGGAG	289	59.1
Si-actin-2	ACGACCATGTTCCCTGGTATT	ATCGTACTCCGCCTTTGAGAT	183	59.0
*Si-actin-2	ATCCAGCCCCTTGTATGTGA	ACGCCCAACAATACTTGGAA	98	60.3
*EF-Iα	CAACAAGATGGATGCCACCAC	GAGATTGGGACGAAGGCAATC	126	59.43

*****primers used for qPCR only
