# Supplementary material for: Phosphate Concentration and Arbuscular Mycorrhizal Colonisation Influence the Growth, Yield and Expression of Twelve PHT1 Family Phosphate Transporters in Foxtail Millet (Setaria italica)
Source: PLoS One. 2014 Sep 24;9(9):e108459. doi: 10.1371/journal.pone.0108459 (PMC4177549; doi:10.1371/journal.pone.0108459)
Supplement: Table S2 — Effect of phosphate on the pigment content of foxtail millet. (PDF) [file pone.0108459.s003.pdf]

Table S2. Effect of phosphate on the pigment content of foxtail millet

Concentration of Phosphate (µM)	Concentration of chlorophyll a (µg/g)	Concentration of chlorophyll b (µg/g)	Concentration of chlorophyll a &b (µg/g)
0.0	109.0 ± 22.5 e	123.5 ± 37.3 a	232.6 ± 46.9 c
10	179.8 ± 20.4 b	65.9 ± 6.2 c	245.8 ± 26.4 b
50	223.1 ± 21.7 a	77.5 ± 6.7 b	300.6 ± 28.4 a
100	116.3 ± 22.7 d	23.7 ± 9.5 d	140.0 ± 30.1 d
300	123.8 ± 12.9 c	7.5 ± 5.0 e	131.3 ± 17.6 e

Chlorophyll content was estimated on 5th week after initiating the experiment. Third leaf from the bottom of each plant was used for pigment extraction. Data shown are means with standard deviation bars (n = 5). Different letters denote significant differences between values with in that column as determined by a Bonferroni post hoc test.
