# Supplementary material for: Phosphate Concentration and Arbuscular Mycorrhizal Colonisation Influence the Growth, Yield and Expression of Twelve PHT1 Family Phosphate Transporters in Foxtail Millet (Setaria italica)
Source: PLoS One. 2014 Sep 24;9(9):e108459. doi: 10.1371/journal.pone.0108459 (PMC4177549; doi:10.1371/journal.pone.0108459)
Supplement: Table S3 — Gene and protein details of foxtail millet PHT1 phosphate transporters and reference genes. (DOCX) [file pone.0108459.s004.docx]

**Table S3. Gene and protein details of foxtail millet *PHT1* phosphate transporters and reference genes**

| **Name of the PHT1 gene** | **Gene locus name** | **Genomic location** | **Gene size**  **(bp)** | **Number of introns** | **UniProt accession number** | **Protein**  **length (AA)** | **Protein molecular mass (Da)** |
| --- | --- | --- | --- | --- | --- | --- | --- |
| *SiPHT1;1* | Si035047m.g | scaffold_9: 56064180 to 56065811 | 1632 | Nil | K4A841 | 543 | 58,962 |
| *SiPHT1;2* | Si035059m.g | scaffold_9: 18406229 to 18408135 | 1907 | Nil | K4A853 | 541 | 58,781 |
| *SiPHT1;3* | Si011707m.g | scaffold_7: 10369609 to 10371219 | 1611 | Nil | K3YBW3 | 536 | 58,757 |
| *SiPHT1;4* | Si035143m.g | scaffold_9: 56061035 to 56062880 | 1846 | Nil | K4A8D7 | 527 | 57,815 |
| *SiPHT1;5* | Si014865m.g | scaffold_6: 35853438 to 35855063 | 1626 | Nil | K3YKU6 | 541 | 58,444 |
| *SiPHT1;6* | Si038747m.g | scaffold_9: 56770072 to 56771774 | 1703 | 1 | K4AIN5 | 530 | 57,072 |
| *SiPHT1;7* | Si008326m.g | scaffold_4: 39294165 to 39295799 | 1635 | 1 | K3Y2A4 | 539 | 58,164 |
| *SiPHT1;8* | Si033125m.g | scaffold_2: 10990282 to 10991907 | 1626 | Nil | K4A2M5 | 541 | 58,435 |
| *SiPHT1;9* | Si004288m.g | scaffold_5: 31290689 to 31292488 | 1800 | 1 | K3XQV1 | 564 | 61,346 |
| *SiPHT1;10* | Si011876m.g | scaffold_7: 10503463 to 10505907 | 2445 | 3 | K3YCD1 | 578 | 63,557 |
| *SiPHT1;11* | Si012482m.g | scaffold_7: 10612771 to 10614417 | 1647 | 1 | K3YE23 | 509 | 55,713 |
| *SiPHT1;12* | Si006153m.g | scaffold_4: 16273297 to 16276625 | 3329 | 1 | K3XW45 | 567 | 61,232 |
| *Siactin-2* | Si026509m.g | scaffold_8: 3363186 to 3365709 | 2524 | 3 | K3ZIV6 | 377 | 41,694 |
| *EF-Iα* | Si022040m.g | scaffold_3: 23407018 to 23410156 | 3139 | 1 | K3Z668 | 447 | 49,266 |
