# Supplementary material for: Phosphate Concentration and Arbuscular Mycorrhizal Colonisation Influence the Growth, Yield and Expression of Twelve PHT1 Family Phosphate Transporters in Foxtail Millet (Setaria italica)
Source: PLoS One. 2014 Sep 24;9(9):e108459. doi: 10.1371/journal.pone.0108459 (PMC4177549; doi:10.1371/journal.pone.0108459)
Supplement: Table S4 — Cycle threshold (Ct) values of Siactin-2 and EF-Iα in qPCR analysis. (DOCX) [file pone.0108459.s005.docx]

**Table S4. Cycle threshold (Ct) values of *Siactin-2* and *EF-Iα* in qPCR analysis**

| **Hydroponics experiment** | | |  | **Mycorrhiza experiment** | | |
| --- | --- | --- | --- | --- | --- | --- |
| **Sample type and growth condition** | **Name of the gene** | **C_T_ value (mean*)** |  | **Sample type and growth condition** | **Name of the gene** | **C_T_ value (mean*)** |
| Leaf 300 μM Pi | *Siactin-2* | 35.87 |  | Leaf non-AM | *Siactin-2* | 33.13 |
| Leaf 10 μM Pi | *Siactin-2* | 36.59 |  | Leaf AM | *Siactin-2* | 33.48 |
| Root 300 μM Pi | *Siactin-2* | 36.67 |  | Root non-AM | *Siactin-2* | 36.83 |
| Root 10 μM Pi | *Siactin-2* | 34.14 |  | Root AM | *Siactin-2* | 37.27 |
|  | | |  |  | | |
| Leaf 300 μM Pi | *EF-Iα* | 29.74 |  | Leaf non-AM | *EF-Iα* | 29.07 |
| Leaf 10 μM Pi | *EF-Iα* | 29.45 |  | Leaf AM | *EF-Iα* | 30.01 |
| Root 300 μM Pi | *EF-Iα* | 30.09 |  | Root non-AM | *EF-Iα* | 31.66 |
| Root 10 μM Pi | *EF-Iα* | 29.83 |  | Root AM | *EF-Iα* | 30.89 |

*=3 replicates
