# Supplementary material for: Phosphate Concentration and Arbuscular Mycorrhizal Colonisation Influence the Growth, Yield and Expression of Twelve PHT1 Family Phosphate Transporters in Foxtail Millet (Setaria italica)
Source: PLoS One. 2014 Sep 24;9(9):e108459. doi: 10.1371/journal.pone.0108459 (PMC4177549; doi:10.1371/journal.pone.0108459)
Supplement: Table S5 — Plant PHT1 sequences employed for phylogenetic analysis shown in Figure 3 . (DOCX) [file pone.0108459.s006.docx]

**Table S5. Plant PHT1 sequences employed for phylogenetic analysis shown in Figure 3**

| **Species** | **Name** | **UniProt accession** | **Species** | **Name** | **UniProt accession** |
| --- | --- | --- | --- | --- | --- |
| *Astragalus sinicus* | AsPHT1;1 | K0I2D6 | *Brachypodium distachyon* | BdPHT1;1 | I1GKJ5 |
| *A. sinicus* | AsPHT1;2 | K0ID45 | *B. distachyon* | BdPHT1;2 | I1GYQ3 |
| *A. sinicus* | AsPHT1;3 | K0I9L6 | *B. distachyon* | BdPHT1;3 | I1H271 |
| *A. sinicus* | AsPHT1;4 | K0IIN5 | *B. distachyon* | BdPHT1;4 | I1H9N9 |
| *A. sinicus* | AsPHT1;5 | K0I855 | *B. distachyon* | BdPHT1;5 | I1H9P0 |
| *Glycine max* | GmPHT1;1 | C3UZD3 | *B. distachyon* | BdPHT1;7 | I1HQ08 |
| *G. max* | GmPHT1;2 | C3UZD4 | *B. distachyon* | BdPHT1;8 | I1I033 |
| *G. max* | GmPHT1;3 | I1L7H9 | *B. distachyon* | BdPHT1;9 | I1I4C3 |
| *G. max* | GmPHT1;4 | C3UZD2 | *B. distachyon* | BdPHT1;10 | I1IW10 |
| *G. max* | GmPHT1;5 | C0LZ80 | *B. distachyon* | BdPHT1;11 | I1IW12 |
| *G. max* | GmPHT1;6 | E8Z9A4 | *B. distachyon* | BdPHT1;12 | I1IW13 |
| *G. max* | GmPHT1;7 | I1N9Q5 | *B. distachyon* | BdPHT1;13 | I1IW14 |
| *G. max* | GmPHT1;8 | C3UZD0 | *Hordeum vulgare* | HvPHT1;1 | Q8H6E0 |
| *G. max* | GmPHT1;9 | E8Z9A7 | *H. vulgare* | HvPHT1;2 | Q84LH9 |
| *G. max* | GmPHT1;10 | E8Z9A8 | *H. vulgare* | HvPHT1;4 | Q84JQ1 |
| *G. max* | GmPHT1;11 | E8Z9A9 | *H. vulgare* | HvPHT1;5 | Q84VI7 |
| *G. max* | GmPHT1;12 | C3UZD6 | *H. vulgare* | HvPHT1;6 | Q8H6D9 |
| *G. max* | GmPHT1;13 | E8Z9B1 | *H. vulgare* | HvPHT1;7 | Q84VI6 |
| *G. max* | GmPHT1;14 | C3UZD5 | *H. vulgare* | HvPHT1;8 | Q6Y3A2 |
| *Lotus japonicus* | LjPHT1;1 | Q1T6Z7 | *H. vulgare* | HvPHT1;9 | C7C2W8 |
| *L. japonicus* | LjPHT1;2 | Q1T6Z6 | *H. vulgare* | HvPHT1;10 | C7C2W9 |
| *L. japonicus* | LjPHT1;3 | Q1T6Z8 | *H. vulgare* | HvPHT1;11 | K0J6Y2 |
| *L. japonicus* | LjPHT1;4 | B5RHV8 | *H. vulgare* | HvPHT1;12 | M0XNV3 |
| *Medicago truncatula* | MtPHT1;1 | O22301 | *Oryza brachyantha* | ObPHT1;1 | J3LK23 |
| *M. truncatula* | MtPHT1;3 | A5H2U5 | *O. brachyantha* | ObPHT1;3 | J3N2Z3 |
| *M. truncatula* | MtPHT1;4 | Q8GSG4 | *O. brachyantha* | ObPHT1;7 | J3LJR1 |
| *M. truncatula* | MtPHT1;5 | A5H2U6 | *O. brachyantha* | ObPHT1;8 | J3N2Z4 |
| *M. truncatula* | MtPHT1;6 | B2G3Q0 | *O. brachyantha* | ObPHT1;12 | J3LK20 |
| *M. truncatula* | MtPHT1;7 | G7I3V6 | *O. brachyantha* | ObPHT1;13 | J3LVU1 |
| *Populus trichocarpa* | PtPHT1;1 | B9HU93 | *O. brachyantha* | ObPHT1;A | J3KTY9 |
| *P. trichocarpa* | PtPHT1;2 | B9NBW8 | *Oryza sativa* subsp. *japonica* | OsPHT1;1 | Q8H6H4 |
| *P. trichocarpa* | PtPHT1;3 | B9NBW6* | *O. sativa* | OsPHT1;2 | Q8GSD9 |
| *P. trichocarpa* | PtPHT1;4 | B9H8L3 | *O. sativa* | OsPHT1;3 | Q7XDZ7 |
| *P. trichocarpa* | PtPHT1;5 | B9GS18 | *O. sativa* | OsPHT1;4 | Q8H6H2 |
| *P. trichocarpa* | PtPHT1;6 | B9H7C7 | *O. sativa* | OsPHT1;5 | Q7X7V2 |
| *P. trichocarpa* | PtPHT1;7 | B9H8L4 | *O. sativa* | OsPHT1;6 | Q8H6H0 |
| *P. trichocarpa* | PtPHT1;8 | B9N1T7* | *O. sativa* | OsPHT1;7 | Q8H6G9 |
| *P. trichocarpa* | PtPHT1;9 | B9GPP2 | *O. sativa* | OsPHT1;8 | Q8H6G8 |
| *P. trichocarpa* | PtPHT1;10 | B9N991 | *O. sativa* | OsPHT1;9 | Q8H6G7 |
| *P. trichocarpa* | PtPHT1;11 | B9H5V9 | *O. sativa* | OsPHT1;10 | Q69T94 |
| *P. trichocarpa* | PtPHT1;12 | B9GFU1 | *O. sativa* | OsPHT1;11 | Q94DB8 |
| *Arabidopsis thaliana* | AtPHT1;1 | Q8VYM2 | *O. sativa* | OsPHT1;12 | Q8H074 |
| *A. thaliana* | AtPHT1;2 | Q96243 | *O. sativa* | OsPHT1;13 | Q7XRH8 |
| *A. thaliana* | AtPHT1;3 | O48639 | *Setaria italica* | SiPHT1;1 | K4A841 |
| *A. thaliana* | AtPHT1;4 | Q96303 | *S. italica* | SiPHT1;2 | K4A853 |
| *A. thaliana* | AtPHT1;5 | Q8GYF4 | *S. italica* | SiPHT1;3 | K3YBW3 |
| *A. thaliana* | AtPHT1;6 | Q9ZWT3 | *S. italica* | SiPHT1;4 | K4A8D7 |
| *A. thaliana* | AtPHT1;7 | Q494P0 | *S. italica* | SiPHT1;5 | K3YKU6 |
| *A. thaliana* | AtPHT1;8 | Q9SYQ1 | *S. italica* | SiPHT1;6 | K4AIN5 |
| *A. thaliana* | AtPHT1;9 | Q9S735 | *S. italica* | SiPHT1;7 | K3Y2A4* |
| *Poncirus trifoliata* | PtaPHT1;1 | K9MVW0 | *S. italica* | SiPHT1;8 | K4A2M5 |
| *P. trifoliata* | PtaPHT1;2 | K9MW71 | *S. italica* | SiPHT1;9 | K3XQV1 |
| *P. trifoliata* | PtaPHT1;3 | K9MX45 | *S. italica* | SiPHT1;10 | K3YCD1* |
| *P. trifoliata* | PtaPHT1;4 | K9MW18 | *S. italica* | SiPHT1;11 | K3YE23 |
| *P. trifoliata* | PtaPHT1;7 | K9MW74 | *S. italica* | SiPHT1;12 | K3XW45 |
| *Capsicum frutescens* | CfPHT1;1 | A0T3D2 | *Sorghum bicolor* | SbPHT1;1 | C5XFB2 |
| *C. frutescens* | CfPHT1;2 | A0T3D4 | *S. bicolor* | SbPHT1;2 | C5YCS2 |
| *C. frutescens* | CfPHT1;3 | A0T3D6 | *S. bicolor* | SbPHT1;3 | C5YCS5 |
| *C. frutescens* | CfPHT1;4 | A0T3D9 | *S. bicolor* | SbPHT1;A | C5WXW0 |
| *C. frutescens* | CfPHT1;5 | A0T3E2 | *S. bicolor* | SbPHT1;B | C5X0E3 |
| *Nicotiana tabacum* | NtPHT1;1 | Q9ST22 | *S. bicolor* | SbPHT1;C | C5X0E4 |
| *N. tabacum* | NtPHT1;1.1 | Q9AYT3 | *S. bicolor* | SbPHT1;D | C5WXV9 |
| *N. tabacum* | NtPHT1;2 | Q9AYT2 | *S. bicolor* | SbPHT1;E | C5YMT5 |
| *N. tabacum* | NtPHT1;2.1 | Q9AYT1 | *S. bicolor* | SbPHT1;F | Q1KSA4 |
| *N. tabacum* | NtPHT1;3 | A0T3D8 | *S. bicolor* | SbPHT1;G | C5X4D7 |
| *N. tabacum* | NtPHT1;4 | A0T3E1 | *S. bicolor* | SbPHT1;H | C5Z1R1 |
| *N. tabacum* | NtPHT1;5 | A0T3E4 | *Triticum aestivum* | TaPHT1;1 | Q93WQ9 |
| *Petunia hybrida* | PhPHT1;1 | A7KTC5 | *T. aestivum* | TaPHT1;2 | Q93WR0 |
| *P. hybrida* | PhPHT1;3 | B2CPI5 | *T. aestivum* | TaPHT1;3 | AK333026.1# |
| *P. hybrida* | PhPHT1;4 | B2CPI6 | *T. aestivum* | TaPHT1;8 | Q5CC72 |
| *P. hybrida* | PhPHT1;5 | B2CPI7 | *T. aestivum* | TaPHT1;10 | K0J8S4 |
| *Solanum lycopersicum* | SlPHT1;1 | O22548 | *T. aestivum* | TaPHT1;11 | K0J4E5 |
| *S. lycopersicum* | SlPHT1;2 | O22549 | *T. aestivum* | TaPHT1;12 | K0J492 |
| *S. lycopersicum* | SlPHT1;3 | Q5MJA7 | *Zea mays* | ZmPHT1;1 | Q49B46 |
| *S. lycopersicum* | SlPHT1;4 | Q563I3 | *Z. mays* | ZmPHT1;2 | Q49B45 |
| *S. lycopersicum* | SlPHT1;5 | K4C5X5 | *Z. mays* | ZmPHT1;3 | Q49B44 |
| *S. lycopersicum* | SlPHT1;A | K4C4P4 | *Z. mays* | ZmPHT1;4 | Q49B43 |
| *S. lycopersicum* | SlPHT1;B | K4CUR6 | *Z. mays* | ZmPHT1;5 | Q49B42 |
| *Solanum melongena* | SmePHT1;1 | A0T3D3 | *Z. mays* | ZmPHT1;6 | Q5CC71 |
| *S. melongena* | SmePHT1;2 | A0T3D5 | *Z. mays* | ZmPHT1;7 | B6TXX9 |
| *S. melongena* | SmePHT1;3 | A0T3D7 | *Z. mays* | ZmPHT1;A | B6TJ37 |
| *S. melongena* | SmePHT1;4 | A0T3E0 | *Z. mays* | ZmPHT1;B | C4JC09 |
| *S. melongena* | SmePHT1;5 | A0T3E3 | *Z. mays* | ZmPHT1;C | K7TNA5 |
| *Solanum tuberosum* | StPHT1;1 | Q43650 | *Z. mays* | ZmPHT1;D | H9C1D0 |
| *S. tuberosum* | StPHT1;2 | M1B1M4 |  |  |  |
| *S. tuberosum* | StPHT1;3 | Q8W4W9 |  |  |  |
| *S. tuberosum* | StPHT1;4 | Q5ICC1 |  |  |  |
| *S. tuberosum* | StPHT1;5 | M0ZQS5 |  |  |  |
| *S. tuberosum* | StPHT1;A | M1AM99 |  |  |  |

Gene names are taken where possible from the literature. For those genes not so described, they have been arbitrarily designated A, B etc.

*UniProt sequence likely to be erroneous and so replaced by modified sequence predicted from examination of the gene sequence. #GenBank accession of mRNA encoding the protein designated TaPHT1;3.
